# Enzymatically Driven Mineralization of a Calcium–Polyphosphate Bleaching Gel

**DOI:** 10.3390/bioengineering11010083

**Published:** 2024-01-16

**Authors:** Mariangela Ivette Guanipa Ortiz, Yendry Regina Corrales Ureña, Flávio Henrique Baggio Aguiar, Débora Alves Nunes Leite Lima, Klaus Rischka

**Affiliations:** 1 Department of Restorative Dentistry, Piracicaba Dental School, University of Campinas—UNICAMP, Piracicaba 13414-903, Brazil; 2 Faculty of Production Engineering, University of Bremen, Am Fallturm 1, 28359 Bremen, Germany; 3 National Laboratory of Nanotechnology LANOTEC—National Center of High Technology CeNAT, 1.3 Km North of the United States Embassy, San José 1174-1200, Costa Rica; 4 Fraunhofer Institute for Manufacturing Technology and Advanced Materials IFAM, 28359 Bremen, Germany

**Keywords:** biomimetic, tooth bleaching, polyphosphates, alkaline phosphatase, hydrogen peroxide, circular dichroism

## Abstract

To examined alkaline phosphatase enzyme (ALP) activity and the effects of incorporating it in the thickener solution of a hydrogen-peroxide-based bleaching gel containing calcium-polyphosphate (CaPP) on the orthophosphate (PO_4_^3−^) levels, bleaching effectiveness, and enamel microhardness. ALP activity was assessed at different pH levels and H_2_O_2_ concentrations, and in H_2_O- and Tris-based thickeners. Circular dichroism (CD) was used to examine the ALP secondary structure in water-, Tris-, or H_2_O_2_-based mediums. The PO_4_^3−^ levels were evaluated in thickeners with and without ALP. Enamel/dentin specimens were allocated into the following groups: control (without bleaching); commercial (Whiteness-HP-Maxx); Exp-H (H_2_O-based); CaPP-H; ALP-H (CaPP+ALP); Exp-T (Tris-based); CaPP-T; and ALP-T (CaPP+ALP). Color changes (ΔE/ΔE00) and the bleaching index (ΔWID) were calculated, and surface (SMH) and cross-sectional microhardness (CSMH) were assessed. The two-way ANOVA and Tukey’s post-hoc tests were used to compare ALP and PO_4_^3−^ levels; generalized linear models were used to examine: ΔE/ΔE00/SMH/CSMH; and Kruskal–Wallis and Dunn’s tests were used for ΔWID (α = 5%). The ALP activity was higher at pH 9, lower in H_2_O_2_-based mediums, and similar in both thickeners. The CD-spectra indicated denaturation of the enzyme upon contact with H_2_O_2_. The PO_4_^3−^ levels were higher after incorporating ALP, and the ΔE/ΔE00/ΔWID were comparable among bleached groups. SMH was lower after bleaching in Exp-H, while CSMH was highest in ALP-T.

## 1. Introduction

The emergence of new biomimetic materials is accompanied by an increase in their use for the purpose of remineralizing dental hard tissues [1,2]. Calcium–polyphosphate sub-microparticles (CaPP), which are amorphous polymers containing ±40 phosphate units linked by phosphoanhydride bonds, have previously been synthetized using co-precipitation [3]. CaPP can act as a precursor for the formation of crystalline materials that repair altered dental hard tissues, with the orthophosphates’ (PO_4_^3−^) releasement from the polymers acting as a building block for enamel remineralization [4,5].

Upon the completion of polymerization, the PO_4_^3−^ and calcium divalent cations (Ca^2+^) in the CaPP become unavailable for mineralization. For this, polyphosphates deliver phosphates to the mineralization sites, preventing “off-site” mineral precipitation [6,7]. CaPP can release ions through the following: (1) spontaneous hydrolytic degradation of the polyphosphate chain at a slower rate; (2) cleavage of the chain by a phosphatase enzyme (e.g., alkaline phosphatase [ALP]) at an accelerated rate, especially in mediums with higher pH levels (e.g., buffer solutions) [7,8,9]. The latter method ensures precise apatite saturation at the mineralization site [6].

Much of the evidence on hydrogen-peroxide-based (HP) bleaching gels to date has focused on the preservation of their clinical effectiveness while reducing their main adverse effects, such as dental sensitivity and defects in hard tissue surfaces [10,11,12]. Although some experimental HP bleaching gels have reported promising results after the incorporation of chemical activators [13,14] or remineralizing agents [15,16,17,18], evidence in this field remains unclear, necessitating the development and assessment of new materials [19,20].

**Figure 1:**
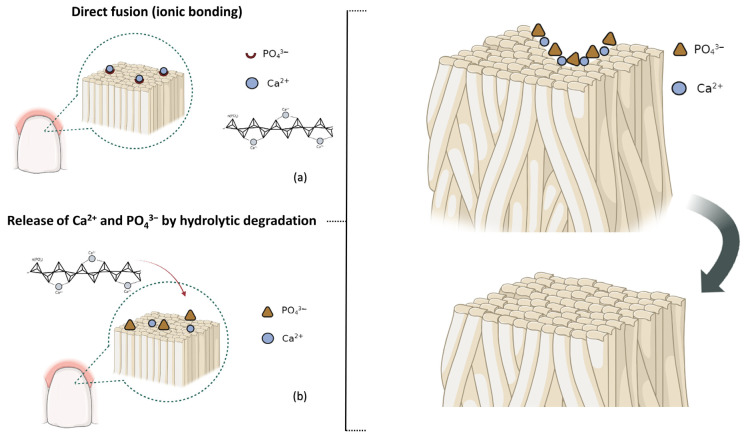
Schematic description of the remineralization mechanism of the calcium polyphosphate (CaPP) by direct integration with the tooth surface via ionic bonding (**a**), and/or through the release of Ca^2+^ and PO_4_^3−^ by hydrolytic degradation, facilitating the biomineralization of the dental tissues through the transformation of the amorphous precursors into crystalline materials (**b**).

The CaPP has the advantage of being a biocompatible compound that can integrate into the tooth surface and release Ca^2+^ and PO_4_^3−^, facilitating the biomineralization of the dental tissues through the transformation of the amorphous precursors into crystalline materials (Figure 1) [3,18,21]. This process is biologically upregulated by the enzymatic activity of ALP [22,23,24]. This boosting of CaPP degradation is a biological process that, within a remineralizing–bleaching system, could facilitate enamel bleaching and the concurrent preservation of its physical properties. However, as ALP activity is pH-dependent [25], the properties of such a bleaching system should be assessed in both water and buffer thickener solutions (e.g., Tris-buffer) that do not interfere with the H_2_O_2_ action.

The objective of the current study was to assess ALP activity and examine its effects on PO_4_^3−^ levels in the thickener solution of a highly concentrated, hydrogen-peroxide-based bleaching gel containing CaPP (HP-CaPP-ALP). Furthermore, its bleaching efficacy and its effect on enamel microhardness after in vitro treatment were also examined. The research hypotheses were as follows: (1) ALP activity will be higher in the presence of Tris and alkaline mediums but decrease in the presence of H_2_O_2_; (2) the PO_4_^3−^ levels of the HP-CaPP-ALP (thickener compound) will be higher than that of gels without ALP; and (3) color changes (ΔE; ΔE00; and ΔWID) and the microhardness of enamel treated with HP-CaPP-ALP will be similar to or superior to that of enamel treated with experimental and commercial 35%-HP gels without CaPP and ALP.

## 2. Materials and Methods

### 2.1. Assessment of ALP Activity and Its Secondary Structure Using Circular Dichroism Spectroscopy (CD)

The activity of ALP derived from calf intestine (524572; Merck KGaA, Darmstadt, Germany) was assessed under different conditions, including Tris-HCl buffers with varying pH levels (six, seven, eight, nine, and ten); different concentrations of H_2_O_2_ (i.e., 7, 4, and 0.6 mg/mL); and thickeners prepared with different mediums (i.e., Tris-based and H_2_O-based). Assessments were carried out using colorimetric endpoint assays (ALP-Kit; Labtest-Diagnóstico-S.A., Lagoa Santa, Brazil), and all analyses were performed within 10 or 30 min of incorporating ALP into the medium of choice. At this point, 250 µL of a buffer solution was added to 25 µL of a thymolphthalein monophosphate solution and kept under 700 rpm agitation (Thermomixer Comfort; Eppendorf-SE, Hamburg, Germany) at 37 °C (2 min). Thereafter, 25 µL of the sample was mixed with the solution and stored under the previously mentioned conditions for 10 min, and then 1000 µL of a color reagent was added to react with the hydrolyzed thymolphthalein and modify the final color of the product [16]. The ALP absorbance values were determined using a microplate reader at 590 nm (Infinite-200-PRO; Tecan, Mannedorf, Switzerland), and then converted into units per liter (U/L) according to the manufacturer’s formula, as follows:

ALP = (Asamp/Astand) × 45(1)

where Asamp represents the absorbance of the sample and Astand represents the absorbance of the standard.

Structural changes in the ALP according to the medium that was used (i.e., MilliQ water; Tris-buffer; HP-35%-H (water-based); HP-35%-T (Tris-based); and Sol-B (Tris-based)) were characterized by circular dichroism (CD) spectra using an Applied Photophysics Chirascan spectrometer running the Pro-Data Chirascan software (v4.2.22). At least three scans were recorded for each sample under the following conditions: temperature = 25 °C; wavelength range of 190–250 nm using intervals of 1 nm in Suprasil quartz cells (Hellma U.K. Ltd., Southend-on-Sea, UK), with a path length of 0.02 mm. The mean values of the scans were then calculated, and the respective baseline values were subtracted. The resulting net spectra were smoothed using a Savitzky–Golay filter with smoothing windows of 5–10 data points.

The mean residue ellipticity (ΘMRE) was defined as follows:

ΘMRE = Θ/(c × n × l)

where Θ is the raw CD ellipticity (mdeg); n is the number of amino acids in the solvated peptide; l is the path length of the quartz cuvette that was used (mm); and c is the molar concentration of the peptides. The CD spectra were analyzed using the BeStSel web server to allow for an estimation of the relative amount of specific secondary conformational elements in the samples.

Intestinal-type alkaline phosphatase solution in 6 mM Tris/HCI, 6 mM magnesium chloride, 0.12 mM zinc chloride, and 40% glycerol pH 7.6 (Calbiochem, Darmstadt, Germany; activity: 30,100.0 U/mL; molecular weight: 140,000 Da) were used. A total of 60 μL of each solution was added to the enzyme (concentration 17.8 mg/mL) and left to react for 15 min before carrying out measurements.

### 2.2. Assessment of PO_4_^3−^ Levels

Orthophosphate (PO_4_^3−^) levels in the thickener component of bleaching systems containing 0.5 wt% of CaPP in H_2_O or Tris-HCl-buffer were assessed, with or without ALP incorporation. A colorimetric Phosphate Assay Kit (MAK-308; Sigma-Aldrich, Saint Louis, MO, USA) was used [26] to examine: CaPP-H (H_2_O-based/CaPP); ALP-H (H_2_O-based/CaPP+ALP); CaPP-T (Tris-based/CaPP); and ALP-T (Tris-based/CaPP+ALP). The thickener was weighed (25 mg), and 15 U of exogenous ALP from calf intestine (524572; Merck KGaA, Darmstadt, Germany) was added and kept in an incubator (700 rpm) at 37 °C (Thermomixer Comfort 5355; Eppendorf SE, Hamburg, Germany). Thereafter, the gels were further diluted to a final concentration of 1.6 mg/mL so that they remained within the assay kit detection range (0.4–50 µM PO_4_^3−^).

The prepared samples were mixed with malachite green in a 96-well-plate and kept in an incubator (KS260; IKA^®^, Staufen, Germany) under agitation (250 rpm) at 37 °C. The optical densities of the solutions were read in triplicate at 620 nm using a microplate reader (Infinite 200 PRO; Tecan Trading, AG, Männedorf, Switzerland). The PO_4_^3−^ concentration was determined by employing a calibration curve obtained from a series of aqueous solutions containing known concentrations of PO_4_^3−^ (40, 32, 24, 16, 12, 8, 4, 0 µM), as per the manufacturer’s instructions [27]. Moreover, thickeners without CaPP or ALP were used as blank controls and subtracted from the group’s final values.

### 2.3. Enamel Preparation and Bleaching Treatment

After assessing the orthophosphate levels, an in vitro bleaching treatment of bovine enamel specimens was carried out. Power calculations for the color (effect size = 0.35; α = 0.05; β = 0.80; correlation = 0.5) and microhardness analyses (effect size = 0.35; α = 0.05; β = 0.80; correlation = 0.2) were based on previous evidence, and the findings showed a minimum sample size of 10 per group per analysis (GPower 3.1-software; Heinrich-Heine-Universität, Germany).

A bench drill (FSB16; Schulz, Joinville, Brazil) was used to create 200 enamel–dentin disks (5.7 × 2.3 mm) from bovine incisors previously disinfected with 0.1% Thymol solution. The specimen surfaces were regularized using granulated silicon carbide paper (600, 1200, 2500, and 4000 grid) and polished using felt and diamond pastes (1 and 1/4 µm), mounted in a polishing unit (Arotec S.A. Ltd.; Cotia, Brazil) [28]. The prepared specimens were divided into two groups for color and microhardness analyses. The color specimens were stained by immersing them in a black tea solution (1.6 g of black tea in 100 mL of boiled distilled water for 5 min), which was renewed daily for 6 days, followed by one week of immersion in artificial saliva.

After an initial color and surface microhardness (SMH) assessment (12% deviation considering a 359KHN mean), the specimens were randomized into eight groups (*n* = 10) including control (without bleaching); commercial (Whiteness-HP-Maxx, FGM); Exp-H (water-based-thickener without CaPP); CaPP-H (0.5 wt%-CaPP); ALP-H (0.5 wt%-CaPP+ALP); Exp-T (Tris-based-thickener without CaPP); CaPP-T (0.5 wt%-CaPP); and ALP-T (0.5 wt%-CaPP+ALP; Table 1).

The specimens were then subjected to three bleaching sessions at weekly intervals, with each session including three applications lasting 15 min each. In the ALP-H and ALP-T groups, ALP was first mixed with the thickener solutions and left to react for 10 min, in order to allow for it to react with the CaPP before denaturation upon contact with H_2_O_2_. Thereafter, similar to the other groups, an H_2_O_2_-based solution was mixed with the thickener (3:1 weight proportion), and 0.02 g of the bleaching gel was applied to the enamel surface. The specimens were stored in artificial saliva (1.5 mM Ca^2+^, 0.9 mM P, 150 mM KCl, 0.05 µg F−/mL, and 0.1 M Tris-buffer at pH 7) at 37 °C throughout the bleaching process and up to 14 days after the completion of treatment [18].

### 2.4. Color (ΔE, ΔE00, ΔWID) Assessment

To determine the bleaching effectiveness of the products, the dental/enamel specimens had their color assessed using a calibrated spectrophotometer (CM 700d; Minolta, Tokyo, Japan) in a standardized light environment (GTI Mini Matcher MM-1; GTI, Cedar Rapids, IA, USA). Three readings were recorded at baseline, after the 1st, 2nd, and 3rd sessions, and 7 and 14 d after the completion of bleaching, and the spectral distribution was quantified using the CIELab color space. For the five assessment points (T1 = after 1st session; T2 = after 2nd session; T3 = after 3rd session; T4 = 7 days after bleaching; T5 = 14 days after bleaching), the total color change (ΔE/ΔE00) and bleaching indices (ΔWID) [29,30] were calculated using the following formulas, with baseline readings serving as the reference:

ΔE = [(ΔL*)^2^ + (Δa*)^2^ + (Δb*)^2^] ^^1^/_2_^ (2)

ΔE00 = [(ΔL′/KLSL)^2^ + (ΔC′/KCSC)^2^ + (ΔH′/KHSH)^2^ + RT (ΔC′/KCSC) (ΔH′/KHSH)] ^^1^/_2_^ (3)

ΔWID = 0.511ΔL* − 2.324Δa* − 1.100Δb*(4)

where: ΔL = assessment time L*—baseline L*; Δa = assessment time a*—baseline a*; Δb = assessment time b*—baseline b*. As per the CIEDE2000 metric, ΔL′, ΔC′, and ΔH′ represent changes in brightness, chrome, and hue values, respectively. SL, SC, and SH are parameters that regulate the values of the coordinates; KL, KC, and KH are correction parameters for the experimental conditions; and RT takes into account the interaction between differences in chroma and hue in the blue region [30].

### 2.5. Surface Microhardness (SMH) and Cross-Sectional Microhardness (CSMH) Assessment

Enamel SMH was assessed using a microdurometer (FM-100; Future-Tech-Corp, Kanagawa, Japan) at baseline (prior to 1st bleaching session) and again after completion of the third bleaching session. A Knoop indenter (25 gf/5 s) was used to make five indentations 100 μm apart [31].

To assess the in-depth microhardness of the enamel, after the completion of bleaching treatment, the specimens were first cut longitudinally using a double-faced diamond disk, and one of the sections was immersed in epoxy resin (2001; Redelease, São Paulo, SP, Brazil). Grinding and polishing were carried out as described previously to expose the specimen surface. A Knoop indenter (25 gf/5 s) was used to create three columns (spaced at least 100 µm apart) of indentations at depths of 10, 20, 40, 60, 80, 100, 120, 140, 160, 180 µm from the enamel surface to allow for the measurement of CSMH using a microdurometer [28,32].

### 2.6. Statistical Analyses

After descriptive and exploratory analyses, the two-way ANOVA and Tukey’s post-hoc tests were used to compare ALP and PO_4_^3−^ levels according to pH and H_2_O_2_ concentration. A *t*-test (GraphPad-Software-Prism-8; Boston, MA, USA) was used to compare ALP levels according to type of thickener. Generalized linear models (GLM) were used to examine the ΔE and ΔE00, while ΔWID was analyzed using the Kruskal–Wallis and Dunn’s tests. SMH was analyzed using a mixed GLM model adjusted for repeated measures in time, while CSMH was analyzed using a GLM model, considering the effects of bleaching treatment with subdivided plots in the depths (R Core Team; Computing, Vienna, Austria). All analyses were carried out at a significance level of 5%.

## 3. Results

### 3.1. Activity and Secondary Structure of ALP

**Figure 2:**
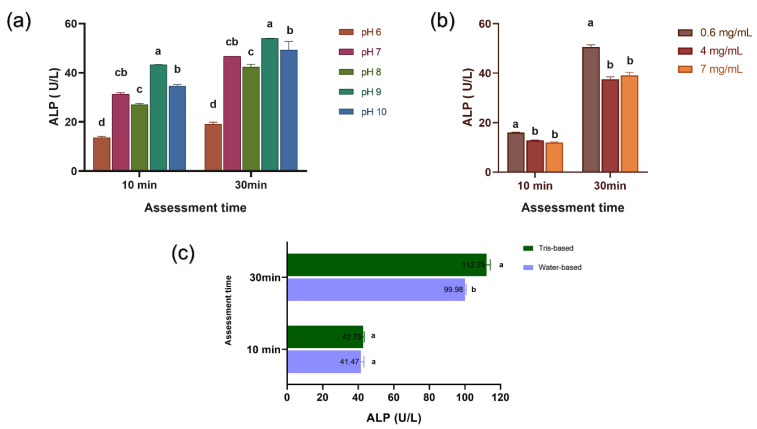
Alkaline phosphatase (ALP) activity, as determined by the spectrophotometric technique (590 nm) after 10 and 30 min of assessment in: (**a**) different pH levels; (**b**) different concentrations of hydrogen peroxide; and (**c**) thickeners based on water or Tris. The lowercase letter indicates a statistically significant difference between the groups.

The lowest and highest ALP activities were observed at pH levels of 6 (*p* < 0.04) and 9, respectively, after 10 and 30 min of incubation (*p* < 0.009; Figure 2a). In H_2_O_2_-containing solutions, the highest ALP activity was observed at a concentration of 0.6 mg/mL (*p* < 0.01), regardless of the time (Figure 2b). Both thickeners exhibited a similar ALP activity after 10 min; however, the Tris-based medium was seen to exhibit a higher ALP activity after 30 min (*p* = 0.0006; Figure 2c).

**Figure 3:**
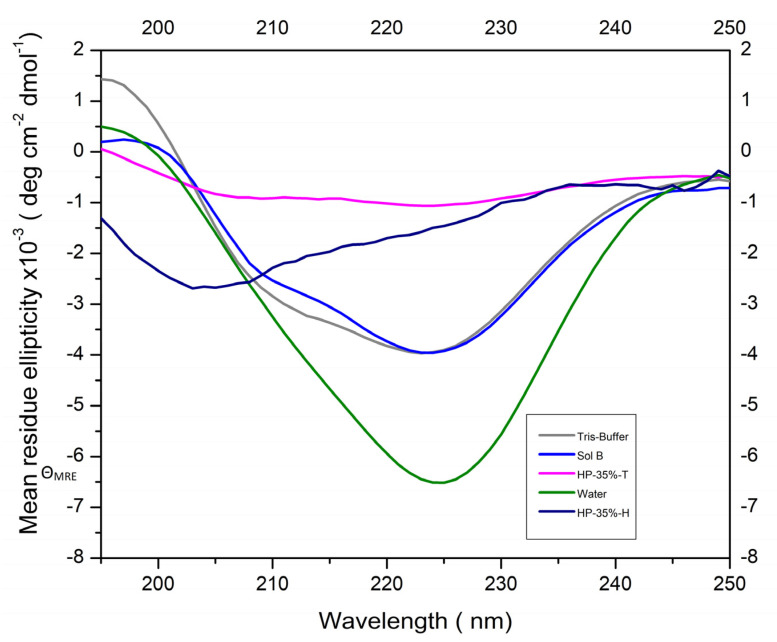
CD spectra of the alkaline phosphatase enzyme (ALP) dispersed in the different solutions.

Significant changes in the CD spectra were observed in the HP-35%-H and HP-35%-T groups, with the negative centered at 225 nm decreasing. Furthermore, a single negative band at 205 nm was present in the HP-35%-H group (Figure 3), and a decrease in the α-Helix (%) of samples diluted with peroxides was observed (Table S1).

### 3.2. PO_4_^3−^ Levels

**Figure 4:**
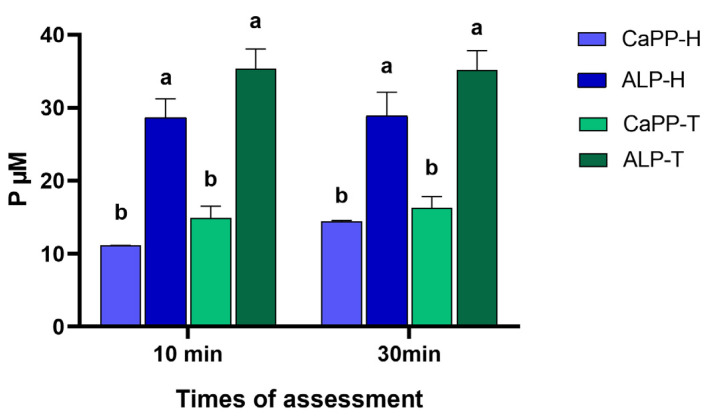
PO_4_^3−^ concentrations (P µM) in the thickeners after 10 or 30 min of the ALP incorporation (15 U): CaPP-H (H_2_O-based with 0.5 wt% CaPP); ALP-H (H_2_O-based with 0.5 wt% CaPP + ALP); CaPP-T (Tris-based with 0.5 wt% CaPP); ALP-T (Tris-based with 0.5 wt% CaPP + ALP). The lowercase letter indicates a statistically significant difference between the groups.

The incorporation of ALP significantly increased free PO_4_^3−^ levels (*p* < 0.001) in both water-based (ALP-H) and Tris-based (ALP-T) thickener solutions. However, PO_4_^3−^ levels did not increase significantly upon prolonging incubation time from 10 to 30 min (*p* > 0.32; Figure 4).

### 3.3. Color (ΔE, ΔE00, ΔWID)

**Figure 5:**
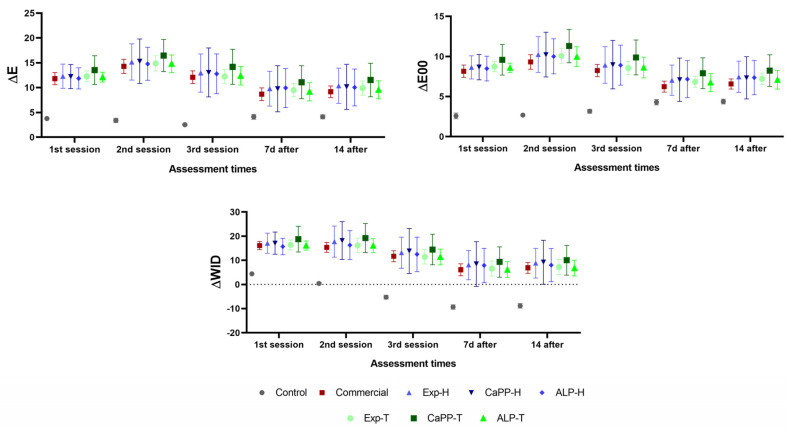
Color variation (ΔE; ΔE00; ΔWID) according to the treatment group and assessment period.

The color difference parameters (ΔE and ΔE00) were higher in the bleached groups compared to the control group (*p* < 0.05), although no statistically significant differences were observed between the commercial and experimental bleaching gels, regardless of composition (*p* > 0.05). Similarly, the ΔWID values were significantly higher in the bleached groups compared to the control group (*p* < 0.05), although no inter-group differences were observed in the former (*p* > 0.05; Figure 5 and Table S2).

### 3.4. Surface Microhardness (SMH)

**Figure 6:**
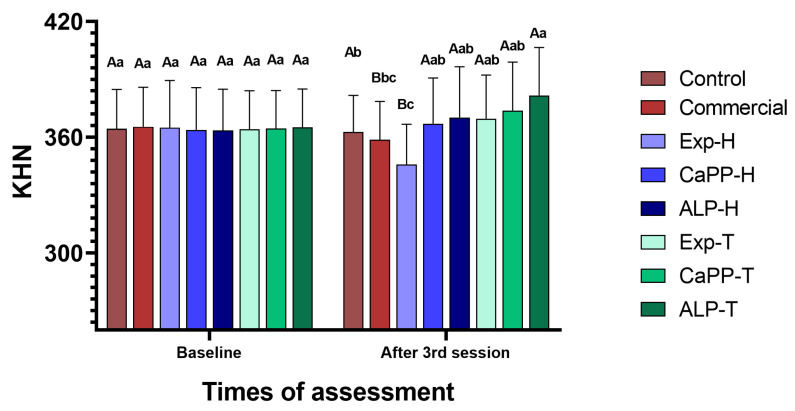
Surface microhardness (SMH) variations according to the treatment group and time of assessment. Different letters indicate significant difference between the assessment times (upper case letters) and the treatment groups (lower case).

All groups exhibited similar SMH values at baseline (*p* > 0.05), although these were seen to be significantly decreased in the Exp-H and commercial groups after the third bleaching session (*p* < 0.05). The ALP-T group exhibited the highest SMH values, and this was significantly different from that of the commercial and control groups (*p*-value < 0.05). In contrast, the Exp-H group exhibited the lowest SMH values, and these were significantly different from that of the CaPP-H, ALP-H, Exp-T, CaPP-T, ALP-T, and control groups (*p* < 0.05; Figure 6).

### 3.5. Cross-Sectional Microhardness (CSMH)

The CSMH values after treatment were significantly higher in the ALP-T group when compared to the other groups (*p* < 0.05). In contrast, the CSMH values were significantly lower in the Exp-H and commercial groups when compared to the control, CaPP-H, CaPP-T and ALP-T groups (*p* < 0.05). Moreover, CSMH was seen to increase up to a depth of 100 µm (*p* < 0.05) and then stabilize in all groups (*p* > 0.05; Table 2).

## 4. Discussion

An examination of the effects of ALP on experimental bleaching gels containing 35%-HP and 0.5 wt% CaPP (HP-CaPP-ALP) showed increased activity in Tris-based and alkaline mediums (pH 9) and decreased activity in the presence of H_2_O_2_. Therefore, the first research hypothesis was accepted. The findings of this study also showed that PO_4_^3−^ levels increased upon the incorporation of ALP; similar color changes (ΔE; ΔE00; ΔWID) were observed in all groups; and the SMH and CSMH values were higher after treatment using gels containing ALP in a Tris-based medium (ALP-T) when compared to the experimental and commercial gels without CaPP. Therefore, the second and third research hypotheses were also accepted.

ALP is a metalloenzyme that catalyzes the hydrolysis of phosphomonoesters such as polyphosphates. It plays a vital role in hard tissue formation due to its increased expression in mineralized tissue cells [22,25], and is use as a relevant indicator of osteoblast differentiation [33,34]. The current study measured ALP activity in different mediums and concentrations, which were chosen based on pilot studies and adjusted to be within the range of activity of a test based on thymolphthalein monophosphate hydrolysis, to enable the identification of the most appropriate method of incorporating it into bleaching gels containing CaPP [16].

H_2_O_2_ was seen to downregulate ALP activity. This was in agreement with the findings of a previous cell analysis [35]. Alkaline mediums with pH nine were considered to be ideal [23], as the ALP catalytic mechanism is favored by the formation of serine phosphate at the enzyme active site, which then reacts with water to form inorganic phosphates [25]. The thickener solutions did not decrease ALP activity and, as a result, both formulas were used for the incorporation of ALP in the subsequent phases of the study. Since the enzyme’s conformational integrity influences its activity [36], the CD spectra support the findings regarding ALP activity. Exposure to H_2_O_2_ induced changes in the secondary structure of the enzyme, as evidenced by the observation of decreased ALP activity in H_2_O_2_-containing solutions.

Although encouraging results have been observed upon incorporating CaPP in bleaching gels containing hydrogen peroxide [18], the addition of ALP can increase the free orthophosphate levels (PO_4_^3−^) and facilitate its subsequent precipitation during bleaching treatment. In this study, the enzymatic effects on PO_4_^3−^ were assessed using a method based on the complexation of triarylmethane dye, malachite green, with phosphatemolybdate ions, as this has greater sensitivity and simplicity [26]. The two-fold-increase in PO_4_^3−^ levels observed in both thickener solutions within 10 min of ALP incorporation suggests scission of the polyphosphate chain, mediated by the exopolyphosphatase. The maintenance of CaPP in an amorphous state can aid this process as it is more susceptible to enzymatic cleavage by ALP [21].

Although the majority of bleaching gels are water-based, previous evidence suggests that Tris-HCl buffers are more suitable when greater ALP activity is required [37] Therefore, two types of thickeners were formulated, and the ALP activity, PO_4_^3−^ levels, bleaching efficacy, and enamel microhardness after treatment were assessed using both. The findings showed that both water- and Tris-based thickeners exhibited similar PO_4_^3−^ levels, even 30 min after incorporation. This could likely be attributed to the fact that higher Ca^2+^ concentrations during CaPP hydrolysis lowered ALP activity up to a certain point, regardless of the medium used [27].

The color changes observed in the specimens confirmed the efficacy of all treatments examined in this study, with the observed values being above the acceptability level (ΔE > 5.4 and ΔE00 > 3.6) [30]. Similarly, the bleaching index (ΔWID) exhibited the highest values when compared to the control group, confirming that CaPP [18] and ALP incorporation did not alter bleaching effectiveness when compared to a commercially established product. Examination after 7 and 14 d showed maintenance of the bleaching effect even after color stabilization. To the best of our knowledge, this is the first study to provide a scientific description of the association between phosphatase and bleaching gel formulations. Based on the initial ALP analysis, it can be inferred that, due to the reduction in its activity in the presence of H_2_O_2_, the last mechanism of action was not altered.

A reduction in SMH was observed after bleaching in the commercial and Exp-H groups, with the latter exhibiting the greatest overall reduction, potentially due to the oxidizing action of H_2_O_2_ [32]. In agreement with previous results [18], the incorporation of CaPP in a bleaching gel was seen to exert a positive effect on enamel properties after treatment, as observed in the CaPP-H and CaPP-T groups. With regards to ALP incorporation, the highest SMH values were observed in the ALP-T and ALP-H groups, suggesting mineral deposition on the surface and/or maintenance of the enamel mineral content potentiated by ALP [5]. Previous studies examining other re-mineralizing agents, such as trimethaphosthate with fluoride or hydrated calcium silicate, suggest that mineral deposition can be effective in preventing surface mineral loss after bleaching [16,17].

The CSMH analysis examined the efficacy of the experimental gels in preventing in-depth mineral loss in the enamel [38], and the findings showed that remineralization was more effective after incorporating CaPP and ALP in Tris-based thickeners (ALP-T), compared to water-based thickeners (ALP-H) or CaPP alone (CaPP-H and CaPP-T). This suggests that the presence of ALP in the water-based thickener was unable to translate into an increase in the release of ions, as reported in the PO_4_^3−^ levels, into the CSMH values. This potentially indicates that the lower pH values of this formula (≈5) induced greater demineralization than that generated by Tris-based thickeners with a more neutral pH (≈6). Therefore, the formerly induced mineral loss that could not be compensated by the ionic burst, unlike the latter, which exhibited CSMH values that surpassed those observed in the control group (without bleaching).

**Figure 7:**
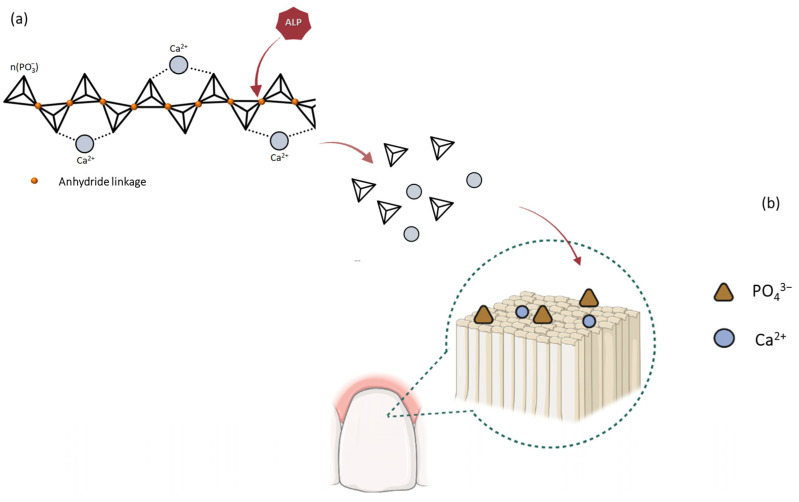
Schematic description of the cleavage of the calcium polyphosphate (CaPP) chain upregulated by the alkaline phosphatase (ALP) within the thickener solution (**a**), and the subsequent release of PO_4_^3−^ and Ca^2+^ from the polyphosphate chain, making them locally available to deposit and interact with the enamel tissue during the bleaching treatment (**b**).

In summary, the hardness values indicate that successful mineral gain and/or preservation is induced by the effect of ALP on CaPP. The upregulation of apatite biomineralization by ALP is initiated by the enzymatic cleavage of one terminal hydrogen–phosphate and one additional proton per anhydride linkage of CaPP [5]. This process releases PO_4_^3−^ and Ca^2+^ from the polyphosphate chain, making them locally available. The precipitation of the ions on the dental surface can result in formation of apatite derived from the amorphous precursors (Figure 7) [39]. The main limitations of the current study were that simulations of other oral environments were not considered and more extensive analyses of the chemical properties of the enamel were not undertaken, and future studies should aim to address these aspects.

Previous studies examining enzymatic action within bleaching gels, with a high H_2_O_2_ concentration (35 wt%), incorporated peroxidase and/or catalase enzymes in HP-based gels and reported promising outcomes with regard to bleaching effectiveness and trans-tissue diffusion [14,40]. However, to the best of our knowledge, this is the first study to demonstrate that a phosphatase enzyme increases ionic availability in a bioinspired way. Moreover, this was also found to be beneficial for the physical properties of the enamel in vitro, likely due to the bioinspired remineralization potential. The elucidation of the biomimetic principles can lead to the development of modified bleaching gels, such as those used in the current study (i.e., containing CaPP and ALP), and facilitate an examination of their effects on the undesirable clinical symptoms associated with dental bleaching treatments.

## 5. Conclusions

Following examination of the effects of incorporating ALP into highly concentrated hydrogen-peroxide-based bleaching gels containing CaPP (HP-CaPP-ALP), the following is concluded:

- ALP activity was higher in mediums with pH 9, reduced in H_2_O_2_ mediums, and remained similar in Tris- or water-based thickeners.
- The PO_4_^3−^ levels were higher following the incorporation of ALP into gel thickener solutions, indicating high polymer scission.
- Both HP-CaPP-ALP (ALP-H and ALP-T) solutions demonstrated adequate bleaching effectiveness.
- The HP-CaPP-ALP gels exhibited increased enamel surface microhardness after treatment when compared to the commercial or experimental gels without CaPP. Moreover, the ALP-T group exhibited the highest microhardness values after treatment.
- The higher PO_4_^3−^ levels in the ALP-T group increased microhardness without decreasing bleaching effectiveness, which suggests a bioinspired remineralization potential.

## Figures and Tables

**Table 1:** Treatment groups for the study, composition, batch/manipulation details.

Treatment Groups	Composition	Batch	Manipulation
Control	No bleaching gel	-	-
Commercial	Glycol, inorganic fillers, H_2_O_2_-30–35 wt%, mixture of pigments, deionized water, thickener.	061222	Mix the components in a 3:1 proportion for 30 s.
Exp-H/Exp-T	Glycerol, propylene glycol, H_2_O_2_-35 wt%, deionized water or Tris buffer solution, acrylic acid thickener.	23/16101	
CaPP-H/CaPP-T	Glycerol, propylene glycol, H_2_O_2_-35 wt%, deionized water or Tris buffer solution, acrylic acid thickener, CaPP 0.5 wt%.	23/16102	
ALP-H/ALP-T	Glycerol, propylene glycol, H_2_O_2_-35 wt%, deionized water or Tris buffer solution, acrylic acid thickener, CaPP 0.5 wt%, ALP (thickener).	23/16103	

**Table 2:** Cross-sectional microhardness (CSMH) values according to the treatment group and distance from the enamel surface.

Distance (µm)/Multiple Comparisons (Distances)	^1^ Group							
	Control	Commercial	Exp-H	CaPP-H	ALP-H	Exp-T	CaPP-T	ALP-T
	Mean (Standard Deviation)							
10/d	253.80 (46.09)	229.27 (48.36)	225.79 (33.32)	261.52 (44.47)	257.23 (63.62)	252.63 (41.45)	268.00 (41.14)	282.98 (44.07)
20/c	315.00 (38.43)	299.19 (42.05)	290.14 (39.99)	327.24 (29.84)	325.07 (56.57)	308.15 (46.48)	338.72 (44.76)	354.99 (18.63)
40/b	357.19 (29.88)	343.43 (25.72)	309.19 (35.97)	352.00 (26.87)	354.71 (47.32)	346.58 (28.99)	354.93 (36.11)	381.47 (20.81)
60/b	356.66 (26.13)	352.95 (26.42)	317.96 (47.54)	359.04 (23.27)	358.61 (58.31)	340.80 (42.03)	359.27 (37.05)	382.85 (19.23)
80/b	363.74 (28.75)	345.53 (29.77)	322.34 (41.43)	352.46 (18.62)	352.47 (57.49)	339.12 (31.32)	362.22 (36.78)	386.80 (19.36)
100/a	370.85 (39.25)	346.59 (30.10)	329.56 (42.04)	359.77 (18.33)	360.73 (62.74)	349.64 (29.56)	367.34 (40.88)	390.13 (15.44)
120/a	370.64 (32.70)	354.94 (25.09)	329.87 (42.09)	369.51 (19.73)	357.56 (58.48)	348.79 (30.21)	367.02 (39.38)	384.07 (15.88)
140/a	371.80 (36.63)	342.79 (31.93)	330.85 (45.03)	370.35 (15.59)	360.98 (61.72)	343.10 (29.79)	368.86 (48.97)	394.55 (18.15)
160/a	374.00 (36.79)	350.06 (39.45)	335.78 (46.73)	364.65 (18.90)	360.43 (63.80)	344.84 (27.62)	370.25 (36.30)	393.73 (12.76)
180/a	371.32 (36.31)	348.36 (38.10)	330.31 (44.89)	369.91 (28.38)	365.69 (61.55)	348.78 (26.32)	365.95 (36.70)	402.54 (16.18)
Multiple comparisons (groups)	B	C	C	B	BC	BC	B	A

^1^ Control (without bleaching); commercial (HP35%-Whitenes HP-Maxx); Exp-H (water-based); CaPP-H (0.5 wt% CaPP); ALP-H (0.5 wt% CaPP + ALP); Exp-T (Tris based); CaPP-T (0.5 wt% CaPP); ALP-T (0.5 wt% CaPP + ALP). p(group) = 0.0025; p(distance) < 0.0001; p(interaction) = 0.2899. Different letters (uppercase in the groups and lowercase in the distance) indicate statistically significant differences (*p* ≤ 0.05)—pooled mean.

## Data Availability

The data presented in this study are available on request from the corresponding author. The data are not publicly available due to privacy.

## Supplementary Materials

The following supporting information can be downloaded at: https://www.mdpi.com/article/10.3390/bioengineering11010083/s1; Table S1: Secondary structure of the alkaline phosphatase (ALP) dispersed in the different solutions; Table S2: Color variation (ΔE; ΔE00; ΔWID) according to the treatment group and assessment period.
