# Supplementary material for: Enzymatically Driven Mineralization of a Calcium–Polyphosphate Bleaching Gel
Source: Bioengineering (Basel). 2024 Jan 16;11(1):83. doi: 10.3390/bioengineering11010083 (PMC10813067; doi:10.3390/bioengineering11010083)
Supplement: Supplementary file 1 [file bioengineering-11-00083-s001.zip › bioengineering-2785701-supplementary.pdf]

Enzymatically-driven mineralization of a calcium-polyphosphate bleaching gel

Supplementary

Table S1. Secondary structure of the alkaline phosphatase (ALP) dispersed in the different solutions

Solution	α-Helix (%)	β-sheets (%)	Turn and coil (%)	NRMSD
Water	11.5	52.7	35.4	0.06
Tris buffer	7.5	28.1	64.4	0.04
Sol B	9.1	26.1	64.8	0.05
HP-35%-T	3.7	27.3	69.1	0.11
HP-35%-H	2.9	28.8	67.4	0.08

Water (MilliQ water); Sol B (Tris-based); HP-35%-T (tris-based); HP-35%-H (water-based); NRMSD: normalized root mean square error deviation

Table S2. Color variation (ΔE; ΔE_{00}; Δ_{WID}) according to the treatment group and assessment period

	**Control**	**Commercial**	**Exp-H**	**CaPP-H**	**ALP-H**	**Exp-T**	**CaPP-T**	**ALP-T**	**p-value**
				**Median (minimum and maximum value)**					
ΔE1	3.62 **B** (2.69; 4.70)	11.44 **A** (9.64; 15.11)	12.45 **A** (7.30; 20.33)	11.69 **A** (8.94; 21.38)	11.30 **A** (8.24; 18.71)	12.51 **A** (10.34; 15.18)	13.11 **A** (8.66; 23.20)	12.25 **A** (9.96; 14.16)	<0.0001
ΔE2	3.59 **B** (2.34; 4.18)	14.05 **A** (11.16; 17.67)	14.36 **A** (7.66; 24.82)	13.19 **A** (9.79; 31.17)	14.31 **A** (9.14; 26.15)	14.64 **A** (12.21; 19.16)	16.20 **A** (10.20; 26.95)	14.65 **A** (11.55; 19.78)	<0.0001
ΔE3	2.46 **B** (2.18; 3.09)	11.61 **A** (9.38; 15.68)	12.28 **A** (5.64; 23.33)	11.18 **A** (5.57; 30.39)	11.70 **A** (7.15; 27.20)	11.92 **A** (9.53; 15.16)	14.15 **A** (7.60; 25.38)	12.44 **A** (9.62; 17.57)	<0.0001
ΔE4	4.10 **B** (2.92; 5.61)	8.03 **A** (6.02; 12.53)	9.04 **A** (2.99; 19.52)	8.00 **A** (3.30; 26.35)	8.94 **A** (4.95; 24.29)	9.68 **A** (6.71; 11.93)	10.91 **A** (4.77; 21.86)	9.03 **A** (6.42; 14.44)	<0.0001
ΔE5	4.00 **B** (3.41; 5.30)	8.93 **A** (6.61; 12.52)	9.80 **A** (3.53; 19.84)	8.43 **A** (3.66; 26.35)	9.22 **A** (5.17; 23.48)	10.20 **A** (7.00; 12.49)	11.64 **A** (5.21; 22.23)	9.25 **A** (6.63; 14.89)	<0.0001
$\Delta E_{00}1$	2.43 **B** (1.82; 3.50)	8.08 **A** (6.91; 9.98)	8.35 **A** (5.62; 13.45)	8.14 **A** (6.87; 14.70)	7.90 **A** (5.63; 13.02)	8.65 **A** (7.37; 10.57)	9.28 **A** (6.50; 15.92)	8.39 **A** (7.20; 10.13)	<0.0001
$\Delta E_{00}2$	2.77 **B** (2.00; 3.19)	9.30 **A** (7.27; 11.40)	9.89 **A** (5.34; 16.06)	9.02 **A** (6.80; 20.25)	9.28 **A** (6.08; 17.12)	9.88 **A** (8.67; 12.67)	11.02 **A** (7.38; 17.88)	9.55 **A** (8.03; 13.61)	<0.0001
$\Delta E_{00}3$	3.25 **B** (2.56; 3.56)	7.99 **A** (6.81; 10.58)	8.99 **A** (4.04; 14.61)	7.90 **A** (4.68; 19.78)	7.83 **A** (5.03; 17.34)	8.46 **A** (6.99; 10.38)	10.11 **A** (5.83; 16.53)	8.45 **A** (6.56; 12.65)	<0.0001
$\Delta E_{00}4$	4.31 **B** (3.62; 5.04)	6.03 **A** (4.95; 8.51)	6.97 **A** (2.84; 12.06)	5.82 **A** (3.92; 17.08)	6.25 **A** (4.07; 15.40)	7.06 **A** (5.31; 8.07)	7.76 **A** (4.31; 14.08)	6.47 **A** (5.09; 10.34)	0.0016
$\Delta E_{00}5$	4.27 **B** (3.80; 5.00)	6.43 **A** (5.37; 8.50)	7.46 **A** (3.11; 12.28)	6.04 **A** (4.22; 17.10)	6.49 **A** (4.27; 14.86)	7.49 **A** (5.58; 8.51)	8.31 **A** (4.50; 14.33)	6.68 **A** (5.31; 10.94)	0.0004

	Control	Commercial	Exp-H	CaPP-H	ALP-H	Exp-T	CaPP-T	ALP-T	p-value
	Median (minimum and maximum value)								
ΔWID1	4.49 **B** (2.29; 5.94)	16.03 **A** (12.38; 20.03)	15.55 **A** (10.94; 31.82)	15.86 **A** (10.19; 34.37)	15.10 **A** (10.24; 26.82)	15.51 **A** (12.11; 20.94)	17.52 **A** (9.71; 36.98)	15.73 **A** (11.99; 20.24)	0.0003
ΔWID2	0.56 **B** (-1.25; 1.32)	14.69 **A** (11.10; 19.61)	15.07 **A** (8.47; 35.52)	14.22 **A** (7.80; 46.18)	15.57 **A** (7.94; 37.92)	16.13 **A** (10.63; 23.17)	18.45 **A** (8.52; 39.41)	16.28 **A** (11.18; 23.89)	0.0003
ΔWID3	-4.89 **B** (-7.78; -4.04)	11.08 **A** (7.43; 17.60)	11.12 **A** (3.55; 30.82)	9.79 **A** (1.10; 47.45)	10.80 **A** (3.81; 38.65)	11.17 **A** (5.70; 18.32)	13.33 **A** (4.96; 35.49)	10.96 **A** (4.77; 19.84)	0.0003
ΔWID4	-8.98 **B** (-12.24; -7.61)	5.43 **A** (1.84; 12.83)	6.30 **A** (-1.38; 25.13)	4.27 **A** (-3.35; 41.87)	6.34 **A** (-0.12; 34.02)	6.52 **A** (0.19; 13.45)	7.89 **A** (-0.13; 30.11)	6.06 **A** (-0.60; 14.91)	0.0003
ΔWID5	-8.45 **B** (-11.30; -7.19)	6.58 **A** (2.77; 12.71)	7.58 **A** (-0.63; 25.57)	4.97 **A** (-2.72; 42.05)	6.84 **A** (0.35; 33.26)	7.30 **A** (0.72; 14.20)	8.79 **A** (1.62; 30.49)	6.20 **A** (0.23; 15.52)	0.0003

Times: ΔE1; $\Delta E_{00}1$; ΔWID1: 1st session. ΔE2; $\Delta E_{00}2$; ΔWID2: 2nd session. ΔE3; $\Delta E_{00}3$; ΔWID3: 3rd session. ΔE4; $\Delta E_{00}4$; ΔWID4: 7 days' after. ΔE5; $\Delta E_{00}5$; ΔWID5: 14 days' after. Groups: Control (saliva only); Commercial (HP35%-Whitenes HP-Maxx); Exp-H (Water based); CaPP-H (0.5 wt% CaPP); ALP-H (0.5 wt% CaPP + ALP); Exp-T (Tris based); CaPP-T (0.5 wt% CaPP); ALP-T (0.5 wt% CaPP + ALP). *Different case letters indicate significant difference between the groups.*
